# Supplementary material for: Identification of photoperiod-induced specific miRNAs in the adrenal glands of Sunite sheep (Ovis aries)
Source: Front Vet Sci. 2022 Jul 22;9:888207. doi: 10.3389/fvets.2022.888207 (PMC9354845; doi:10.3389/fvets.2022.888207)
Supplement: Supplementary file 1 [file Data_Sheet_1.ZIP › Supplementary file/miRDeep2 file.zip!/miRDeep2 file/Know_Structure/SPLP42A1/oar-mir-665.pdf]

miRBase precursor : oar-mir-665
Total read count : 63
oar-miR-665-5p read count : 4
oar-miR-665-3p read count : 55
remaining reads : 4

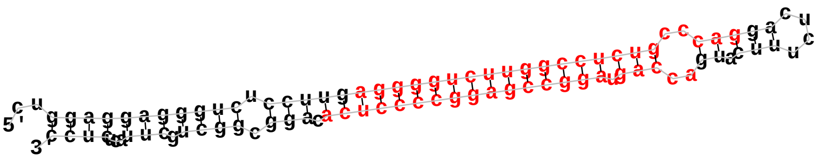

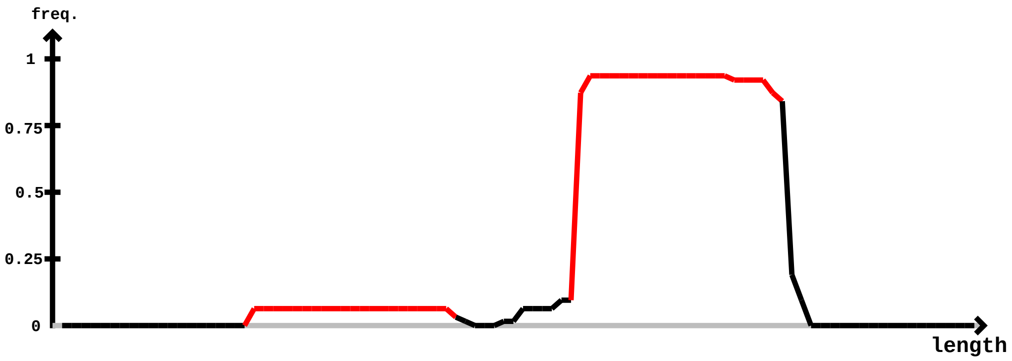


```
                                                   oar-miR-665-3p
                  oar-miR-665-5p
5'- cuggaggagggucuccuugaggggucuuggccucugcccaggacuuucaugaccaguaggccgaggccccucacaggcggcugcuuacucucc   -3'   exp
    ..((((((((((.((((((((((((((((((((((..(((((....))).))..))).))))))))))))))).))).)))).))).))))..    reads   mm   sample
    ...................aggggucuuggccucugccca.....................................................    2       0    seq
    ...................aggggucuuggccucugcccag....................................................    1       0    seq
    ...................aggggucuuggccucugcccagg...................................................    1       0    seq
    ............................................cuuucaugaccaguaggccgaggccccuca...................    1       0    seq
    .............................................uucaugaccaguaggccgaggc..........................    1       0    seq
    .............................................uucaugaccaguaggccgaggccccu......................    2       0    seq
    .................................................ugaccaguaggccgaggccccu......................    1       0    seq
    .................................................ugaccaguaggccgaggccccuca....................    1       0    seq
    ...................................................accaguaggccgaggccccuc.....................    2       0    seq
    ...................................................accaguaggccgaggccccuca....................    38      0    seq
    ...................................................accaguaggccgaggccccucac...................    4       0    seq
    ...................................................accaguaggccgaggccccucaca..................    5       0    seq
    .....................................................ccaguaggccgaggccccuca...................    1       0    seq
    .....................................................ccaguaggccgaggccccucac..................    2       0    seq
    .....................................................ccaguaggccgaggccccucaca.................    1       0    seq
```
